# Recent Advances in Treatments of Primary Focal Segmental Glomerulosclerosis in Children

**DOI:** 10.1155/2016/3053706

**Published:** 2016-04-18

**Authors:** Kyoung Hee Han, Seong Heon Kim

**Affiliations:** ^1^Department of Pediatrics, Jeju National University School of Medicine, Aran 13gil 15, Jeju-si, Jeju Special Self-Governing Province 63241, Republic of Korea; ^2^Department of Pediatrics, Pusan National University Children's Hospital, 20 Geumo-ro, Mulgeum-eup, Yangsan-si, Gyeongsangnam-do 50612, Republic of Korea; ^3^Research Institute for Convergence of Biomedical Science and Technology, Pusan National University Yangsan Hospital, 20 Geumo-ro, Mulgeum-eup, Yangsan-si, Gyeongsangnam-do 50612, Republic of Korea

## Abstract

Focal segmental glomerulosclerosis (FSGS) is a nephrotic syndrome. Up to around 80% of cases of primary FSGS are resistant to steroid treatment. A large proportion of patients with steroid-resistant FSGS progress to end-stage renal disease. The purpose of treatment is to obtain a complete remission of proteinuria, a necessary step that precedes improved renal survival and reduces the risk of progression to chronic kidney disease. When this is not possible, the secondary goal is a partial remission of proteinuria. Reduction or remission of proteinuria is the most important factor predictive of renal survival. We will review the current updated strategies for treatment of primary FSGS in children, including traditional therapies consisting of corticosteroids and calcineurin inhibitors and novel therapies such as rituximab, abatacept, adalimumab, and fresolimumab.

## 1. Introduction

Idiopathic nephrotic syndrome (INS) is a primary renal disorder defined by the three signs of proteinuria, hypoalbuminemia, and edema. Histopathological categories of INS include minimal change disease, focal segmental glomerulosclerosis (FSGS), and diffuse mesangial proliferation [1]. Based on the response to steroid treatment, two types of INS can be characterized: steroid-sensitive nephrotic syndrome (SSNS), in which the proteinuria resolves, and steroid-resistant nephrotic syndrome (SRNS), in which the proteinuria does not resolve [1]. Most patients with minimal change disease respond well to steroid treatment and, currently, renal biopsy is not routinely performed in patients with SSNS. Therefore, the terminology of minimal change disease has become synonymous with SSNS [1]. However, genetic forms of nephrotic syndrome have been emphasized since the discovery of causative mutations in cases with infantile and juvenile SRNS and familial nephrotic syndrome [1]. Histologic findings in the kidney reveal FSGS in most patients with both syndromic and nonsyndromic forms of SRNS [1]. Therefore, it is important to rule out genetic forms of FSGS beforehand to avoid trying ineffective therapies.

A report of the International Study of Kidney Disease in Children (ISKDC) demonstrated that, among patients who were initial steroid responders and those who were not, 3% and 47.5% had FSGS, respectively [2]. Up to around 80% of cases of primary FSGS are resistant to steroids [3]. A large proportion of patients with steroid-resistant FSGS progress to end-stage renal disease [4]. Therefore, nephrologists are challenged and are struggling to treat patients with FSGS. We will review current strategies for medical treatment of primary FSGS in children, omitting any discussion of genetic forms or relapses of FSGS after kidney transplantation.

## 2. Corticosteroids

Steroid therapy is the most basic and standard treatment [1]. Initial steroid therapy is with oral prednisone at a dose of 60 mg/m^2^ or 2 mg/kg per day with a maximum dose of 60 mg per day for 4 to 6 weeks followed by 40 mg/m^2^ or 1.5 mg/kg every other day for 4 to 6 weeks [2, 5, 6]. Ehrich and Brodehl reported the advantages of a 12-week compared to an 8-week steroid treatment protocol for an initial attack of INS [7]. The cumulative rate of patients with sustained remissions after 2 years was significantly higher after the 12-week course than after the 8-week treatment [7]. Several comparative studies have reported that a longer duration of steroid treatment (3–7 months) after an initial 4–8-week daily steroid treatment followed by an alternative protocol significantly reduced the relapse rate compared with the 8-week protocol [7–10]. An alternative treatment for patients who do not achieve remission of nephrotic syndrome after the initial 4 weeks is to administer an intravenous pulse of methylprednisolone, 30 mg/kg per dose, with a maximum of 1 g every other day for 2 weeks [1, 11]. Two-thirds of initial steroid nonresponders eventually became responders with a 2-week course of methylprednisolone [12].

The “Mendoza” protocol is an aggressive treatment strategy based on high-dose methylprednisolone pulse therapy and oral prednisolone, with or without an alkylating agent, administered for a total of 82 weeks (Table 1) [4, 12]. When this protocol was used to treat cases of SRNS, approximately 65% went into complete remission and only 25% progressed to chronic kidney disease [4]. However, prolonged steroid treatment for nephrotic syndrome causes significant side effects, including growth impairment, obesity, hypertension, cataracts, osteoporosis, immune suppression, diabetes mellitus, psychosis, hirsutism, and striae [11].

## 3. Cyclophosphamide

Treatment with a 12-week course of cyclophosphamide at a single dose of 2 mg/kg per day with a maximum cumulative dose of 168 mg/kg reduced the rate of relapse in steroid dependent nephrotic syndrome (SDNS) [6, 13]. A longer remission was observed when cyclophosphamide treatment was used in combination with steroids compared to cyclophosphamide given alone for SDNS [14]. There is little data indicating that cyclophosphamide is effective in SRNS with FSGS and updated version of the Kidney Disease: Improving Global Outcomes (KDIGO) guidelines (http://kdigo.org/home/glomerulonephritis-gn/) suggests that cyclophosphamide should not be given to children with SRNS [15–17]. However, renal failure progresses less frequently in SRNS patients who are partial responders to cyclophosphamide [18, 19].

Cyclophosphamide can be used as monthly intravenous boluses at a dose of 500 mg/m^2^ for 6 months for infrequently relapsing nephrotic syndrome [20]. There are divergent opinions about intravenous pulse cyclophosphamide for steroid-resistant FSGS [21]. However, intravenous pulse cyclophosphamide has been considered as an adjunctive therapy for steroid-resistant FSGS [22].

## 4. Calcineurin Inhibitors

### 4.1. Cyclosporine

Cyclosporine reduces the relapse rate by 80% in patients with SDNS [23]. However, the patients tend to become cyclosporine dependent just as they did with steroids [1]. The remission rate is significantly higher when the therapeutic dosage of cyclosporine is 4–6 mg/kg/day in two divided doses to maintain a trough level between 60 and 80 ng/mL in patients with SDNS [1, 6, 24]. Some research has demonstrated that cyclosporine in combination with steroids is significantly effective in reducing the relapse rate in patients with SRNS [25, 26]. Another study showed that prolonged combination therapy with cyclosporine and steroids, including intravenous pulses of methylprednisolone, maintained the remission rate at 84% for patients with SRNS [27]. Although relapses were frequent, long-term cyclosporine treatment in children with SRNS reduced the progression of chronic kidney disease by reducing proteinuria [28, 29]. Therefore, the Canadian Society of Nephrology (CSN) recently recommended using a calcineurin inhibitor, either cyclosporine or tacrolimus, following standard therapy with steroids for children with SRNS [6]. In recent years, however, cyclosporine has been found not to work in all FSGS patients because patients with a hereditary background of the disease rarely benefit from cyclosporine therapy [30, 31]. Side effects of cyclosporine include hypertrichosis, gum hypertrophy, hypertension, and nephrotoxicity [11].

### 4.2. Tacrolimus

Tacrolimus has recently been highlighted as an alternative to cyclosporine for SRNS [11]. Tacrolimus at a dose of 0.1–0.2 mg/kg per day divided into two doses was effective and well tolerated in children with SRNS, with a complete remission rate of 81% [32, 33]. A target trough level of 5–7 ng/mL should be recommended for SRNS [6, 34]. Cosmetic side effects such as hypertrichosis and gum hypertrophy are not seen with tacrolimus, but any difference between cyclosporine and tacrolimus in the incidence of nephrotoxicity has not been proven [11]. Other side effects, including tremor, arterial hypertension, and diabetes mellitus, can occur. Recently, the efficacy of multidrug therapy consisting of tacrolimus, mycophenolate mofetil (MMF), mizoribine, or leflunomide in combination with steroids for children with SRNS has been reported [35, 36]. While the relapse rate was significantly decreased after multidrug therapy in children with SRNS, long-term safety has not yet been proven.

## 5. Mycophenolate Mofetil

The CSN does not recommend the widespread use of MMF due to limited cost-effectiveness [6]. However, a retrospective study in children with nephrotic syndrome reported that the combined rate of complete and partial remission for MMF was 67% in SRNS, and for refractory cases, combination therapy with MMF, tacrolimus, and steroids yielded a 75% response rate [37]. Compared to tacrolimus, the response rate for MMF was not superior, but MMF has milder side effects than calcineurin inhibitors [11]. MMF was not inferior to calcineurin inhibitors in preventing relapses but only if administered in high doses [38]. MMF can be an alternative agent in patients with adverse effects due to calcineurin inhibitors [1, 6]. MMF can be given at a dose of 1200 mg/m^2^ per day divided into two doses. Area under the concentration-time curve (AUC) measurements are essential in the use of this drug. Commonly reported adverse effects of MMF include metabolic acidosis, infection, diarrhea, abdominal pain, and hyperlipidemia.

## 6. Mizoribine

Mizoribine inhibits inosine monophosphate synthetase and guanosine monophosphate synthetase, resulting in the inhibition of DNA synthesis and cell division. Mizoribine has fewer side effects than azathioprine, which also inhibits an enzyme required for DNA synthesis [39, 40]. Although the mechanism of action for mizoribine is similar to the more widely used azathioprine and MMF, there is little data as to whether it is effective in maintaining remission in nephrotic syndrome [41]. However, there are some case series reporting successful treatment with combination therapy using mizoribine, tacrolimus, or plasmapheresis for children with refractory nephrotic syndrome in Japan [35, 42]. Mizoribine can be administered at a dose of 3 mg/kg once daily before breakfast [35].

## 7. Renin-Angiotensin System Blockade

The KDIGO Work Group recommended renin-angiotensin system (RAS) inhibition with angiotensin converting enzyme inhibitor (ACE-I) or angiotensin II receptor blockers (ARBs) for children with SRNS [6]. RAS blockade combined with MMF was markedly effective in children and young adults with steroid-resistant FSGS [43]. A comparative study of ACE-I and calcium channel blockers in SRNS demonstrated that both ramipril and verapamil reduced proteinuria in patients with SRNS [44]. Another randomized trial of 45 children (10 with FSGS) with normotensive SRNS suggested that fosinopril significantly reduced proteinuria [45]. RAS blockade is especially effective for those patients with chronic renal insufficiency. Monitoring adverse effects of RAS inhibition, notably hyperkalemia and reduction of glomerular filtration rate, is necessary during treatment [11].

## 8. Galactose

Oral galactose, a monosaccharide sugar, inhibits the circulating permeability activity that is the cause of FSGS [46]. However, currently, there is little evidence that it improves proteinuria in children with FSGS [47]. There are only a few case reports about partial remission after oral galactose therapy at a dose of 0.2 g/kg twice a day as a nontoxic and adjunctive agent for SRNS [48, 49].

## 9. Rituximab

Rituximab is a chimeric monoclonal antibody that inhibits CD20-mediated B lymphocytes [11]. It has been used in hematologic malignancies such as B cell non-Hodgkin lymphoma [50]. Rituximab is administered by two to four intravenous infusions at a dose of 375 mg/m^2^ weekly or biweekly [41]. There are several reports of rituximab therapy being an effective steroid-sparing agent for children with SDNS [51, 52] or SRNS [50, 53, 54]. Rituximab and other immunosuppressants such as cyclosporine or MMF combination therapy for SRNS have been reported to be successful in decreasing the relapse rate [55, 56]. However, some patients with SDNS become rituximab dependent as they do with steroids or calcineurin inhibitors [51]. Anti-rituximab autoantibodies may be a concern after repetitive infusion of rituximab [57]. Therefore, the presence of anti-rituximab autoantibodies should be monitored if a severe infusion reaction occurs and B cell depletion is not observed despite rituximab therapy [57, 58].

## 10. Synthetic Adrenocorticotropin Analog

Injection with adrenocorticotropin (ACTH) was used many decades ago as a therapeutic agent for children with nephrotic syndrome, but it has been replaced by cheaper oral steroids [59]. Response to ACTH among patients with FSGS is about 30%, but ACTH gel may be an alternative treatment option for some patients with SRNS [60, 61].

## 11. Abatacept

Abatacept (cytotoxic T-lymphocyte-associated antigen 4–immunoglobulin fusion protein [CTLA-4–Ig]) is an inhibitor of the T-cell costimulatory molecule B7-1 (CD80) [62]. It is currently approved for the treatment of rheumatoid arthritis. Podocyte B7-1 expression was found in patients with proteinuric kidney [62]. Yu and colleagues reported five patients with FSGS, four patients with rituximab-resistant recurrent FSGS, and one patient with steroid-resistant FSGS, whose high proteinuria resolved after abatacept treatment [62]. The authors explained that abatacept may attenuate *β*1-integrin activation in podocytes and decrease proteinuria in patients with B7-1-positive glomerular disease [62].

## 12. Adalimumab

Adalimumab is a human monoclonal antibody directed against tumor necrosis factor-*α* (TNF-*α*), which triggers an autoimmune response [61]. The Novel Therapies for Resistant FSGS (FONT) Study Group conducted a phase I trial of adalimumab in FSGS [63]. Adalimumab was injected subcutaneously every 2 weeks at a dose of 24 mg/m^2^ with a maximum of 40 mg for 16 weeks (total, nine doses) in 10 patients with resistant FSGS [63]. Adalimumab was well tolerated with no serious side effects and 4 out of 10 patients with proteinuria had it decreased by more than 50% [63]. The FONT Study Group formed the basis for a phase 2 study of adalimumab in resistant FSGS [63].

## 13. Fresolimumab

Fibrosis represents the final common pathway of glomerular damage in FSGS, leading to chronic kidney disease. The experience with a wide range of antifibrotic agents was summarized in a recent review article [61]. Fresolimumab is a recombinant, fully human monoclonal antibody and inhibits the activity of all isoforms of transforming growth factor (TGF-*β*) [64]. Trachtman and colleagues conducted a phase 1, single-dose study of fresolimumab in adults with treatment-resistant FSGS [64]. They reported one case of complete remission and two cases of partial remission of proteinuria among a total of 16 patients with resistant FSGS. Further studies are needed to confirm the efficacy of fresolimumab for the treatment of resistant FSGS.

## 14. Conclusions

FSGS is a heterogeneous disorder and response to treatment is individually diverse. SRNS is an indication for a renal biopsy and genetic study. If the renal biopsy shows FSGS and if no pathogenic mutations in podocyte genes can be identified, efforts to minimize drug toxicity are important in pediatric FSGS. However, nephrologists still depend on traditional treatment approaches based on glucocorticoids plus calcineurin inhibitors. A steroid-based combination therapy with one or two drugs such as calcineurin inhibitors, MMF, or RAS blockade for refractory cases can induce partial remission of proteinuria. Severe FSGS cases experiencing treatment toxicities may try novel therapies such as rituximab, abatacept, adalimumab, or fresolimumab. In the future, further investigations of the disease mechanism will assist in the introduction of novel targeted individual approaches for treating FSGS.

## Figures and Tables

**Table 1:** The “Mendoza” protocol for steroid-resistant nephrotic syndrome in children.

Week	Methylprednisolone, 30 mg/kg	Oral prednisone
1-2	Every other day, 6 doses	None
3–10	Every week, 8 doses	2 mg/kg every other day
11–18	Every 2 weeks, 4 doses	With/without taper
19–50	Every 4 weeks, 8 doses	Slow taper
51–82	Every 8 weeks, 4 doses	Slow taper
